# Autosomal dominant stromal corneal dystrophy associated with a *SPARCL1* missense variant

**DOI:** 10.1038/s41431-024-01687-8

**Published:** 2024-08-21

**Authors:** Freddie L. Braddock, Jessica C. Gardner, Nihar Bhattacharyya, Beatriz Sanchez-Pintado, Marcos Costa, Christina Zarouchlioti, Anita Szabo, Petra Lišková, Michael E. Cheetham, Robert D. Young, Caroline Thaung, Alice E. Davidson, Stephen J. Tuft, Alison J. Hardcastle

**Affiliations:** 1 UCL Institute of Ophthalmology, University College London, London, UK; 2 Department of Paediatrics and Inherited Metabolic Disorders, First Faculty of Medicine, Charles University and General University Hospital in Prague, Prague, Czech Republic; 3 Department of Ophthalmology, First Faculty of Medicine, Charles University and General University Hospital in Prague, Prague, Czech Republic; 4 Structural Biophysics Group, School of Optometry & Vision Sciences, Cardiff University, Cardiff, UK; 5 Department of Corneal and External Eye Disease, Moorfields Eye Hospital, London, UK

**Keywords:** Rare variants, Genetics research

## Abstract

Corneal dystrophies are phenotypically and genetically heterogeneous, often resulting in visual impairment caused by corneal opacification. We investigated the genetic cause of an autosomal dominant corneal stromal dystrophy in a pedigree with eight affected individuals in three generations. Affected individuals had diffuse central stromal opacity, with reduced visual acuity in older family members. Histopathology of affected cornea tissue removed during surgery revealed mild stromal textural alterations with alcianophilic deposits. Whole genome sequence data were generated for four affected individuals. No rare variants (MAF < 0.001) were identified in established corneal dystrophy genes. However, a novel heterozygous missense variant in exon 4 of *SPARCL1*, NM_004684: c.334G > A; p.(Glu112Lys), which is predicted to be damaging, segregated with disease. SPARC-like protein 1 (SPARCL1) is a secreted matricellular protein involved in cell migration, cell adhesion, tissue repair, and remodelling. Interestingly, SPARCL1 has been shown to regulate decorin. Heterozygous variants in *DCN*, encoding decorin, cause autosomal dominant congenital stromal corneal dystrophy, suggesting a common pathogenic pathway. Therefore, we performed immunohistochemistry to compare SPARCL1 and decorin localisation in corneal tissue from an affected family member and an unaffected control. Strikingly, the level of decorin was significantly decreased in the corneal stroma of the affected tissue, and SPARCL1 appeared to be retained in the epithelium. In summary, we describe a novel autosomal dominant corneal stromal dystrophy associated with a missense variant in *SPARCL1*, extending the phenotypic and genetic heterogeneity of inherited corneal disease.

## Introduction

The cornea is the transparent anterior surface of the eye that transmits and focuses light onto the retina. It has three primary layers: the external epithelium, the fibrous stroma, and an internal corneal endothelium [1, 2]. Corneal dystrophies are a group of inherited disorders that can affect any or all of these layers, leading to loss of vision from opacity or oedema. Other symptoms can include sensitivity to light and painful recurrent corneal epithelial breakdown. They are typically bilateral and symmetrical, with a slow progression. The age of onset varies between dystrophies, with a tendency for the recurrence of disease in donor tissue following corneal transplantation. Corneal dystrophies are not usually associated with abnormality of other ocular structures or with systemic disease [3, 4]. Most corneal dystrophies are autosomal dominant, although autosomal recessive and X-linked corneal dystrophies have been described; however, the genetic basis for some presumed dystrophies remains unresolved [3]. Congenital stromal corneal dystrophy (CSCD) is a rare autosomal dominant corneal dystrophy caused by variants in *DCN* [5–12]. Here, we describe the clinical features, histopathology, and genetic cause of a novel autosomal dominant stromal corneal dystrophy, which exhibits clinical similarities to CSCD and may share a common molecular mechanism.

## Materials and methods

### Patient recruitment and clinical examination

Informed consent was obtained from all participants, and the institutional review boards of Moorfields Eye Hospital (13/LO/1084) and University College London (UCL) (22/EE/0090) approved the study, which conformed to the principles of the Declaration of Helsinki. Clinical examination included the Snellen best-corrected visual acuity, slit-lamp biomicroscopy with gonioscopy, corneal imaging by tomography (Pentacam, Oculus Optikgeräte GmbH, Wetzlar, Germany, and MS-39, CSO, Firenze, Italy) and confocal microscopy (HRT II, Heidelberg Engineering GmbH, Heidelberg, Germany). Following pupil dilation, we examined the fundus at the slit lamp by indirect ophthalmoscopy. We conducted a verbal enquiry regarding general health and any potential systemic comorbidity.

### Filtering for rare variants in whole genome sequence data and segregation analyses

**Fig. 1:**
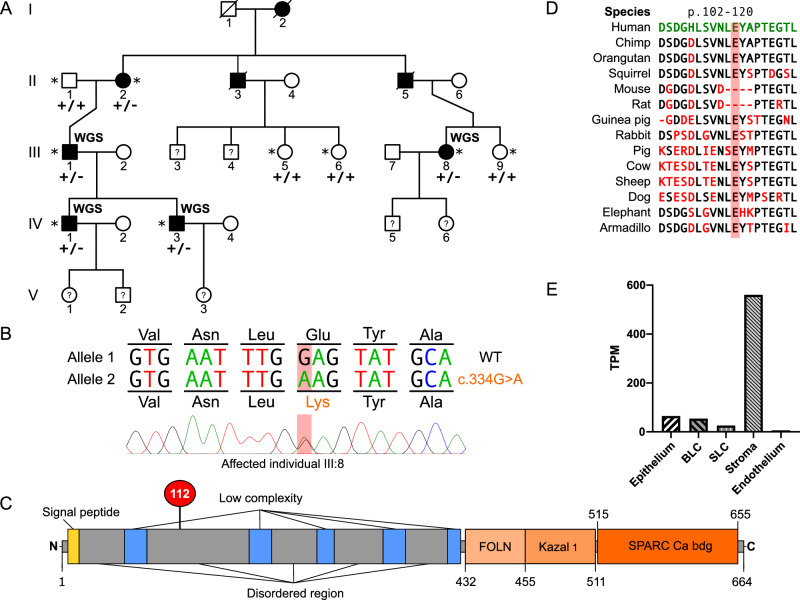
Identification of a novel *SPARCL1* missense variant segregating with autosomal dominant stromal corneal dystrophy. **A** Pedigree structure and segregation of a *SPARCL1* variant. *DNA available. WGS whole genome sequenced. *SPARCL1*, NM_004684: c.334G > A; p.(Glu112Lys), +/− heterozygous variant identified, +/+ wild type on both alleles, verified by PCR amplification and Sanger sequencing. **B** Sanger sequencing chromatogram of *SPARCL1* exon 4 in affected individual III:8 demonstrating a heterozygous c.334G > A. **C** The functional motifs are as follows: signal peptide; FOLN (follistatin/osteonectin-like EGF domain); Kazal 1 (Kazal-type serine protease inhibitor domain); SPARC Ca bdg (secreted protein acidic and rich in cysteine Ca binding region). The disordered regions, parts of the protein lacking definition, are represented by light grey shading. Low-complexity regions are represented in blue shading. p.(Glu112Lys) is located in a disordered region of the protein. Domains are derived from data in Pfam. **D** Conservation of protein sequence across 14 species. **E** RNA-seq transcript expression of *SPARCL1* in the different layers of the cornea. BLC basal limbal crypts, SLC superficial limbal crypts. Data were curated from bulk RNA-seq and presented as transcripts per million (TPM) [19, 20].

We recruited five affected and four unaffected individuals (Fig. 1A) and extracted DNA from peripheral blood leucocytes or saliva using standard protocols. Whole genome sequencing (WGS) was performed on four affected individuals (III:1, III:8, IV:1, IV:3) using the HiSeq 2000 platform (Illumina) (Novogene, Cambridge UK) and 150 base pair paired-end reads with a mean sequencing depth of 34.88. The paired-end reads were mapped to the hg19 reference genome using Burrows–Wheeler Aligner [13]. Single nucleotide variants (SNVs), insertions and deletions (indels) were called using the Genome Analysis Toolkit HaplotypeCaller [14], and structural variants (SVs) and copy number variants (CNVs) were called using Control_FREEC [15] and DELLY [16]. ANNOVAR was used for variant annotation [17]. A genome-wide filtering strategy was adopted after excluding all variant types in all established corneal dystrophy genes (Supplementary Table 1). Heterozygous WGS SNVs and indels shared by the four affected individuals were filtered for a minor allele frequency (MAF) < 0.001 (determined using the gnomAD v3.1.2 database) with CADD >10 [18]. Segregation analysis of candidate variants was performed by PCR and Sanger sequencing following standard protocols. Primer sequences and PCR conditions are described in Supplementary Table 2.

### Haplotype analysis

The WGS data were used for haplotype analysis of a region of interest on chromosome 12 (chr12:91,251,926–91,637,312). SNPs across this region were selected to cover four genes encoding contiguous small leucine-rich proteoglycans *EPYC*, *KERA*, *LUM*, and *DCN*. All SNPs were phased through assumptions of inheritance or where SNPs were present on the same read as follows: SNPs shared between III:1 and his two sons (IV:1 and IV:3) were assumed to have originated from the father’s haplotype; SNPs present in only the two brothers (IV:1 and IV:3) were assumed to have originated from the mother and not from the father’s haplotype; SNPs were also phased if sequencing reads spanned proximal heterozygous SNPs.

### Transcriptomics

Two distinct publicly available corneal RNA-seq datasets were used to determine the expression profile of genes of interest. RNA-seq data were derived from human foetal and adult corneal endothelial cells [19] and four distinct human limbal compartments: the basal limbal crypts, the superficial limbal crypts, the paracentral/central corneal epithelium, and the adjacent limbal stroma [20]. RNA-seq data were aligned to hg19 and hg38 using the Bowtie alignment tool [21]. The featureCounts tool was used to generate gene read counts [22], which were subsequently used to generate transcript per million (TPM) values [23]*.*

### Histopathology

Affected corneal tissue was obtained from individual II:2 following anterior lamellar keratoplasty. It was formalin-fixed and paraffin-embedded (FFPE) following standard protocols. For light microscopy, 4 µm sections were stained with haematoxylin and eosin, periodic acid Schiff, and alcian blue.

### Immunohistochemistry

The excised tissue from affected individual II:2 and normal full-thickness corneal tissue was FFPE and used to localise SPARCL1 and decorin. Using a standard immunohistochemistry (IHC) protocol, 5 µm sections were baked, deparaffinised, and rehydrated using xylene and ethanol gradient washes. Endogenous peroxidase activity was blocked with 0.3% hydrogen peroxide. Samples were incubated for 3 h in citrate solution (Leica, AR9961), at 75 °C for antigen retrieval and later incubated in a blocking solution of 5% donkey serum in 4% Triton in PBS for 30 min. Sections were incubated in primary antibodies goat anti-SPARCL1 (AF2728, R&D Systems, Minneapolis, MN, USA, 1:50) and rabbit anti-DCN (LF-122, Kerafast, Boston, MA, USA, 1:200), at room temperature for 1.5 h then 4 °C overnight. The sections were then washed twice, followed by secondary antibody incubation for 1.5 h at room temperature (donkey anti-Goat A-21432, ThermoFisher, Cambridge, UK, 1:1000, donkey anti-rabbit A-21206, ThermoFisher, Cambridge, UK, 1:1000). Sections were washed and incubated with DAPI (D9564, Sigma-Aldrich, Dorset, UK, 1:2500). Slides were mounted and imaged using Zeiss LSM700, using the same intensity, exposure times, and gain for all sections.

## Results

### Clinical presentation

**Fig. 2:**
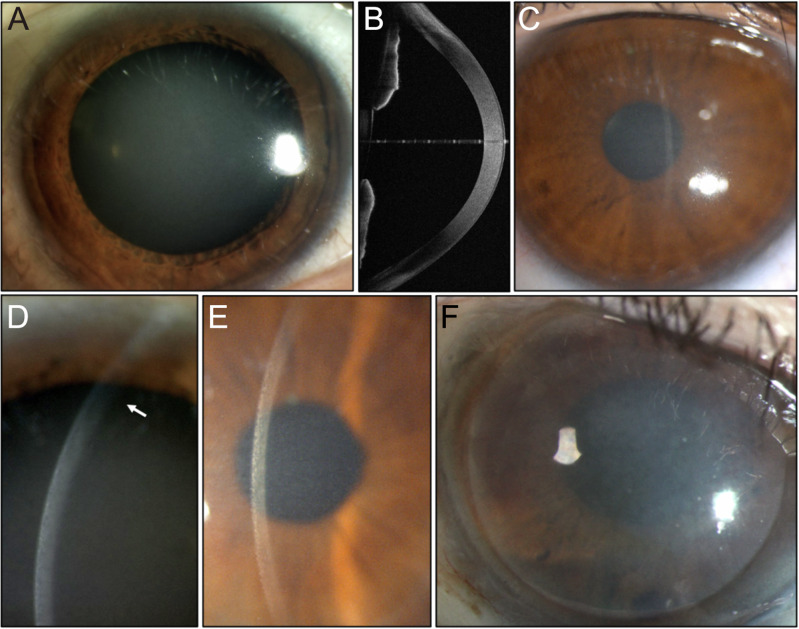
Summary of corneal-specific phenotype. **A** Diffuse illumination of the right cornea of the proband (III:1) at age 63 shows diffuse haze affecting the central corneal stroma. The pupil has been dilated. **B** Ocular coherence tomography scan (III:1) showing the central corneal opacity with no other anterior segment abnormality. **C** The appearance of the cornea of the individual IV:3 at age 30 shows a similar central corneal haze. **D** Slit beam image of the cornea of the individual IV:1 at age 32 years. Haze involves the full thickness of the corneal stroma, but the peripheral cornea is relatively unaffected (arrow). **E** Slit beam image of the individual IV:1 at age 32 years demonstrating the fine granularity of the opacity when seen with retroillumination. **F** Diffuse illumination of the lamellar corneal graft of the right eye of individual II:2 taken ~15 years after surgery. Diffuse corneal haze blurs the details of the pupil margin (arrow), consistent with the recurrence of opacity in the donor tissue.

The pedigree is shown in Fig. 1A. The proband (III:1) is a white male first examined at age 50 years (Fig. 2A, B). At presentation, his corrected visual acuity was 6/5 in each eye, with a refraction of (+2.0/−1.0 × 90) in the right eye and (+1.75/−1.00 × 85) in the left eye. His symptoms were mild visual blur and glare. There was a bilaterally symmetric finely granular central corneal haze without a marked corneal arcus, and no corneal vascularisation or other anterior segment abnormality (Fig. 2A, B). The corneal endothelium also appeared normal, although specular images could not be obtained due to light scatter from the corneal opacity. Confocal microscopy showed extensive stromal remodelling with high interstitial matrix reflectivity (Supplementary Fig. 1). The intraocular pressures were 12 mmHg in each eye, and the central corneal thickness measurements were 594 μm the right and 597 μm the left. He had been prescribed statins, but there was no family history of hypercholesterolaemia, and although he has involuntary head movements, this was not present in other family members. He was last reviewed at age 69 when the opacity had progressed, and his corrected visual acuity was 6/12 in the right eye and 6/24 in the left eye. We examined both sons at ages 34 years (IV:1) and 29 years (IV:3). They were asymptomatic with corrected visual acuities of 6/5 in each eye but with mild bilateral corneal stromal haze without a corneal arcus (Fig. 2C–E). Their ocular examination was otherwise normal. The mother of the proband (II:2) had bilateral anterior lamellar corneal grafts at 62 years in the right eye and 65 years in the left eye, with subsequent cataract extractions at 69 years. She has open-angle glaucoma, but there were no other ocular abnormalities. When examined 18 years after the first corneal transplant, her spectacle-corrected visual acuity was 6/36 on the right and 6/24 on the left with no improvement with pinhole viewing due to mild atrophic macular degeneration. Because there had been a recurrence of haze within the donor corneal stromal tissue (Fig. 2F), a right penetrating keratoplasty was performed at age 88 years. The first cousin of the proband (III:8) was confirmed to have bilateral corneal haze, although the age of onset was unknown. She also had age-related nuclear sclerotic cataracts but with no other ocular abnormality. Her visual acuity is reduced to 6/36 bilaterally, and she is considering combined cataract surgery and corneal transplantation. Individuals I:2, II:3 and II:5 are presumed affected due to familial reports of a corneal haze and visual loss. However, confirmation by clinical examination was not possible. Individuals IV:5, IV:6, V:1, V:2 and V:3 are below 30 years of age; thus, their disease status remains unknown.

### Histology

Light microscopy of the excised anterior lamellar corneal tissue from the proband’s mother (II:2; Fig. 3A, B) showed a normal epithelium and an intact Bowman’s layer. The anterior stroma appeared feathery, with a relatively compact posterior stroma. Alcian blue stain weakly highlighted the anterior stroma without an interlamellar granular distribution.

**Fig. 3:**
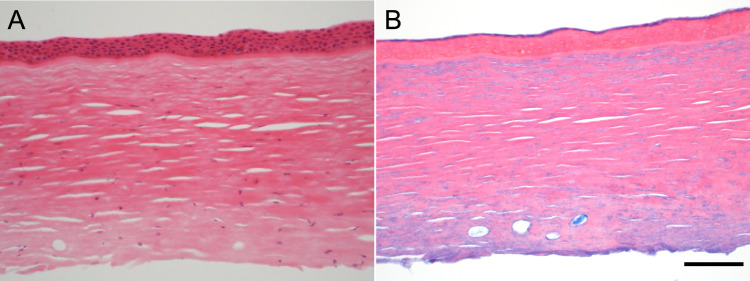
Histology of anterior corneal stroma and epithelium from individual II:2. **A** The epithelium and Bowman’s layer appear normal. The anterior stroma has a feathery appearance, while the posterior stroma has subtle scarring (×20 objective, haematoxylin and eosin). **B** With Alcian blue stain there is weak staining of the anterior stroma in a similar feathery texture (×20 objective).

### Identification of a rare heterozygous *SPARCL1* missense variant

No shared rare (MAF < 0.001) SNVs or indels were identified in the four affected individuals (III:1, III:8, IV:1, IV:3; Fig. 1A) in established corneal dystrophy genes. Similarly, no SVs or CNVs were identified at these loci. This analysis excluded coding and non-coding variants in established corneal genes as candidates for this family. The subsequent filtering for shared rare variants within the genome is shown in Supplementary Fig. 2. The four affected individuals shared 18,092 heterozygous SNV and indel variants with a MAF < 0.001, 29 of which were exonic variants. A final filtering step of CADD >10 revealed 19 variants. Segregation analysis through PCR and Sanger sequencing in the extended family further refined the candidate variant list to four (Supplementary Table 3). The stand-out candidate gene was *SPARCL1* due to high expression levels in corneal tissues (Supplementary Fig. 3) and its biological relevance as a regulator of the protein decorin [24], encoded by the CSCD-associated gene *DCN*. *SPARCL1* is most highly expressed in the stroma (560.28 TPM), followed by the epithelium (64.52 TPM), basal limbal crypts (54.40 TPM), superficial limbal crypts (25.78 TPM), and lastly, the endothelium (0.29 TPM) (Fig. 1E) [19, 20].

The variant of interest is a novel SNV in *SPARCL1* (MAF = 0, gnomAD v3.1.2), predicted as damaging, SIFT = 0 and DANN = 0.998 (dbNSFP version 4.8). This variant is predicted to affect a conserved amino acid in a disordered region of SPARCL1 (Fig. 1C, D), resulting in an amino acid substitution in two gencode transcripts: ENST00000282470.11 exon 4 [NM_004684: c.334G > A; p.(Glu112Lys)], ENST00000418378.5 exon 5 [NM_001128310: c.334G > A; p.(Glu112Lys)], and a non-coding change in the 5′ UTR region of ENST00000503414.5 [NM_001291976.2: c.−42G > A]. SpliceAI revealed a Δ score = 0 across all transcripts, suggesting it is highly unlikely to affect splicing. The level of confidence to infer any effect on protein structure using modelling (Swiss-Model and AlphaFold) were very low (pLDDT < 50) as this is a disordered region of the protein.

### Localisation and expression of SPARCL1 and decorin in control and SPARCL1 p.(Glu112Lys) patient corneal tissue

**Fig. 4:**
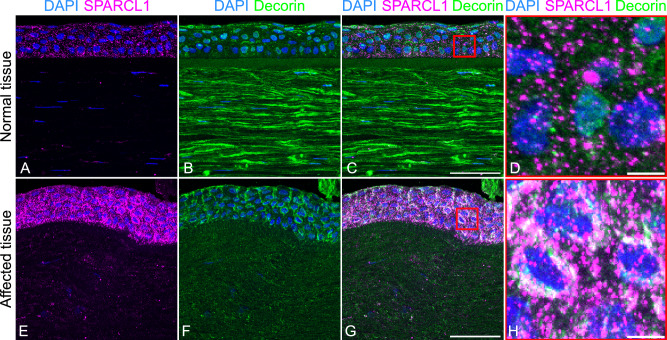
Immunohistochemistry of SPARCL1 and decorin in normal and affected corneal tissue. **A**, **E** Merge of DAPI and SPARCL1 channels show an upregulation of SPARCL1 in the epithelial layer of the affected tissue. **B**, **F** Merge DAPI and decorin channels. The downregulation of decorin is evident in the stroma of the affected tissue. **C**, **G** Merge of all three channels. Scale bars correspond to 50 μm. **D**, **H** Magnification box of (**C**) and (**G**), respectively, scale bars correspond to 5 μm, perinuclear co-localisation of SPARCL1 and decorin is observed in affected tissue.

As SPARCL1 is reportedly involved in the regulation of decorin [24], a protein implicated in CSCD, the localisation of both SPARCL1 and decorin was investigated in control and affected tissue. The control corneal tissue showed high levels of SPARCL1 expression throughout the epithelial layer (Fig. 4A). Within the stroma, SPARCL1 localised in the interlamellar space and within the keratocytes (Fig. 4A). In the affected tissue, whilst the broad localisation of SPARCL1 was relatively unchanged (Fig. 4E), there was an increase in SPARCL1 immunoreactivity within the epithelial layer representing either retention or upregulation (Fig. 4H). There is a striking difference in the levels and localisation of decorin immunoreactivity in the affected tissue compared to control tissue (Fig. 4B, F), with a marked reduction in the corneal stroma. In contrast, the level of decorin immunoreactivity in the epithelial layer was similar to that of the normal cornea (Fig. 4B, F). In contrast to control tissue, SPARCL1 and decorin co-localise in the perinuclear region of epithelial cells in the affected tissue (Fig. 4C, D, G, H), which may represent an interaction site for SPARCL1 and decorin.

### Haplotype analysis of the small leucine-rich proteoglycan region encompassing *DCN*

Although no shared rare variant was identified in the candidate corneal dystrophy gene *DCN*, given the greatly reduced levels of decorin immunoreactivity in the affected cornea we further examined this genomic region to assess if a variant was missed in our WGS analysis pipeline. Haplotype analysis using the WGS data from affected individuals III:1, III:8, IV1, and IV:3 using 11 SNPs as genetic markers between chr12:91,251,926–91,637,312 (Supplementary Fig. 4) revealed that they did not share a haplotype on this region of chr12, which excludes the *DCN* locus as the genetic cause of disease for this pedigree, and suggests that the decorin changes in the corneal stroma are associated with altered SPARCL1 function.

## Discussion

We report the clinical features of five family members with moderate corneal stromal haze, segregating in an apparent autosomal dominant mode of inheritance and presenting as loss of vision but with no other associated ocular or systemic features. The rate of progression of the opacity is gradual, with one individual requiring lamellar corneal transplantation in her 6th decade, with a subsequent penetrating keratoplasty for presumed recurrence of opacity in the donor tissue. The clinical findings, age of onset, and pattern of inheritance distinguished this dystrophy from previously described corneal stromal dystrophies that have prominent focal opacities, such as macular corneal dystrophy [25], granular and lattice corneal dystrophies [26], and fleck corneal dystrophy [27]. However, the appearance of only a diffuse stromal haze resembles Schnyder corneal dystrophy without corneal crystals or significant corneal arcus [28], or CSCD [5, 29]. The age of visual deterioration in this family is later than is characteristic for CSCD (Supplementary Table 2). Following genetic analysis, we excluded all the corneal dystrophies reported in a recent review [3]. However, we identified a novel heterozygous missense variant in exon 4 of *SPARCL1*, NM_004684: c.334G > A; p.(Glu112Lys). This variant is predicted to be damaging, with SIFT = 0 and DANN = 0.998 (dbNSFP version 4.8), and segregates with the disease in the extended family. However, some variant predictors yield different results, such as CADD = 13.71 and REVEL = 0.022 (dbNSFP version 4.8). Discrepancies between variant predictors are typically due to differences in algorithms and training datasets. Several studies have shown that prediction algorithms struggle to classify novel variants [30, 31]. Whilst damaging variants are typically prioritised, benign classification can include disease-causing variants. For instance, an established corneal dystrophy variant in *TGFBI*, NM_000358.3: c.1664G > A; p.(Arg555Gln), has predictions of benign or uncertain classification in several in-silico predictors, SIFT = 0.267, EVE = 0.1704, and REVEL = 0.567 (dbNSFP version 4.8). Therefore, classification should not be used in isolation and several strands of evidence should be used in assessing the pathogenicity of a variant. A common threshold used in filtering strategies, such as CADD >15, may exclude potentially pathogenic variants. Applying this stringent threshold in our study would have filtered out the candidate *SPARCL1* variant.

The SNV in *SPARCL1*, NM_004684: c.334G > A; p.(Glu112Lys), appears novel. We hypothesise this variant acts through gain-of-function or dominant-negative mechanisms. In gnomAD v.3.1.2, there is a rare heterozygous stop-gain variant at the same position, NM_004684: c.334G > T; p.(Glu112Ter) (MAF = 0.00002629). *SPARCL1* is predicted to tolerate loss of function variants (LOEUF = 0.97, gnomAD v.2.1.1). In the recently released gnomAD v.4.1.0, two other rare missense variants alter the same amino acid: NM_004684: c.336G > C; p.(Glu112Asp) (MAF = 6.20e − 7) and NM_004684: c.334G > C; p.(Glu112Gln) (MAF = 6.20e − 7). It is important to note that gnomAD v.4.1.0 contains large biobank data which recruit from the general population. Consequently, individuals with rare and late onset disease may be included in this version of gnomAD, therefore it is uncertain if these missense variants are disease causing.

The *SPARCL1* variant identified in the study family, NM_004684: c.334G > T; p.(Glu112Ter), has a high disease propensity value of 1.14 [32]. In comparison, the alternate missense variants NM_004684: c.336G > C; p.(Glu112Asp) and NM_004684: c.334G > C; p.(Glu112Gln) have lower disease propensity values of 0.48 and 0.59, respectively. This difference in propensity values suggests these alternate variants could be benign.

The identification of a novel variant in *SPARCL1* prompted further investigation into its potential functional significance. SPARCL1 is a matricellular secreted glycoprotein that regulates extracellular matrix (ECM) synthesis and cell–ECM communication, likely in a tissue-specific and ECM environment-dependent manner [24]. A *Sparcl1* −/− knockout mouse model developed corneal haze faster than wild-type mice after superficial corneal injury, and the phenotype could be rescued by administration of exogenous SPARCL1, highlighting the role of *Sparcl1* in corneal wound healing in mice [33]. Additionally, SPARCL1 stimulates decorin production [24]. *DCN* is the established genetic cause of CSCD, with reports of cases with premature stop codons [5–7, 9–12]. Although CSCD typically presents with an onset in childhood, there is at least one report of the onset of the disease in adulthood, in which a novel missense *DCN* variant was identified [8]. Williams et al. reported an increase in corneal decorin expression by immunofluorescence in CSCD patient tissue (NM_001920.5: c.948delA; p.(His317Thrfs*11)) compared to control tissue [10]. In contrast, a mouse model of CSCD (NM_007833.6: c.952delT; p.(Ser318Profs*5)) showed decreased expression of decorin in the cornea compared to control tissue [34]. Collectively, these studies highlight potential species differences in corneal homoeostasis.

*SPARCL1* encodes secreted protein acidic and rich in cysteine-like 1, previously known as hevin. The protein structure and function of SPARCL1 have not been completely characterised. SPARCL1 shares its three functional domains, follistatin-like, Kazal-like and EF-hand, with its paralogous protein SPARC (Fig. 3C). How SPARCL1 regulates decorin is unclear although it has been hypothesised that it may influence decorin availability through interaction and/or stabilisation of the precursor pro-decorin [24]. In our study, the observed change in stromal decorin expression in affected tissue could be explained by constitutive binding of mutant SPARCL1 to pro-decorin, impacting decorin’s availability and interaction with collagen in the ECM. Alternatively, a ternary link between SPARCL1, decorin and collagen could explain how SPARCL1 regulates decorin without direct SPARCL1-decorin binding [24]. Finally, the reduction in decorin levels may not be directly implicated in the pathogenic pathway, but rather a consequence of the disease process.

A limitation of this study is that we have identified only one pedigree with a rare variant in *SPARCL1*. Identifying additional affected individuals with a similar phenotype would further validate *SPARCL1* as a corneal dystrophy gene. The size of the pedigree and access to the patient DNA samples and tissue has been a strength of this study. However, the late onset of the condition means the younger generation of the pedigree is limited to an uncertain diagnosis. If further corneal tissue becomes available, complementary approaches to histopathology and IHC could be used including transcriptomics. A potential limitation for future studies is the challenge of developing a mouse model for this condition (*SPARCL1*, NM_004684: c.334G > A; p.(Glu112Lys)), as rodents lack four amino acids encompassing the residue (Fig. 1D), and may lack biological relevance due to species differences.

In summary, we present the phenotype and genotype of a previously undescribed autosomal dominant corneal stromal dystrophy characterised by slowly progressive corneal opacity with a relatively late onset of visual loss. We hypothesise that the missense variant in *SPARCL1* causes stromal corneal haze through its dysregulation of decorin and, subsequently, dysregulation of the ECM in the corneal stroma. The exact mechanism through which the mutant SPARCL1 results in decorin dysregulation is unclear and future investigations are warranted.

## Supplementary information

SUPPLEMENTAL MATERIAL

## Data Availability

The datasets generated and analysed during the current study are available from the corresponding author on reasonable request. The genotype and phenotype of the associated variant, *SPARCL1*, NM_004684: c.334G > A; p.(Glu112Lys), have been uploaded to GeneMatcher (Submission ID: 94311) and ClinVar (Variation ID: 3257740; Accession: VCV003257740.1).
