# Supplementary material for: Autosomal dominant stromal corneal dystrophy associated with a SPARCL1 missense variant
Source: Eur J Hum Genet. 2024 Aug 21;32(12):1583–9. doi: 10.1038/s41431-024-01687-8 (PMC11607396; doi:10.1038/s41431-024-01687-8)
Supplement: Supplementary file 1 — SUPPLEMENTAL MATERIAL [file 41431_2024_1687_MOESM1_ESM.pdf]

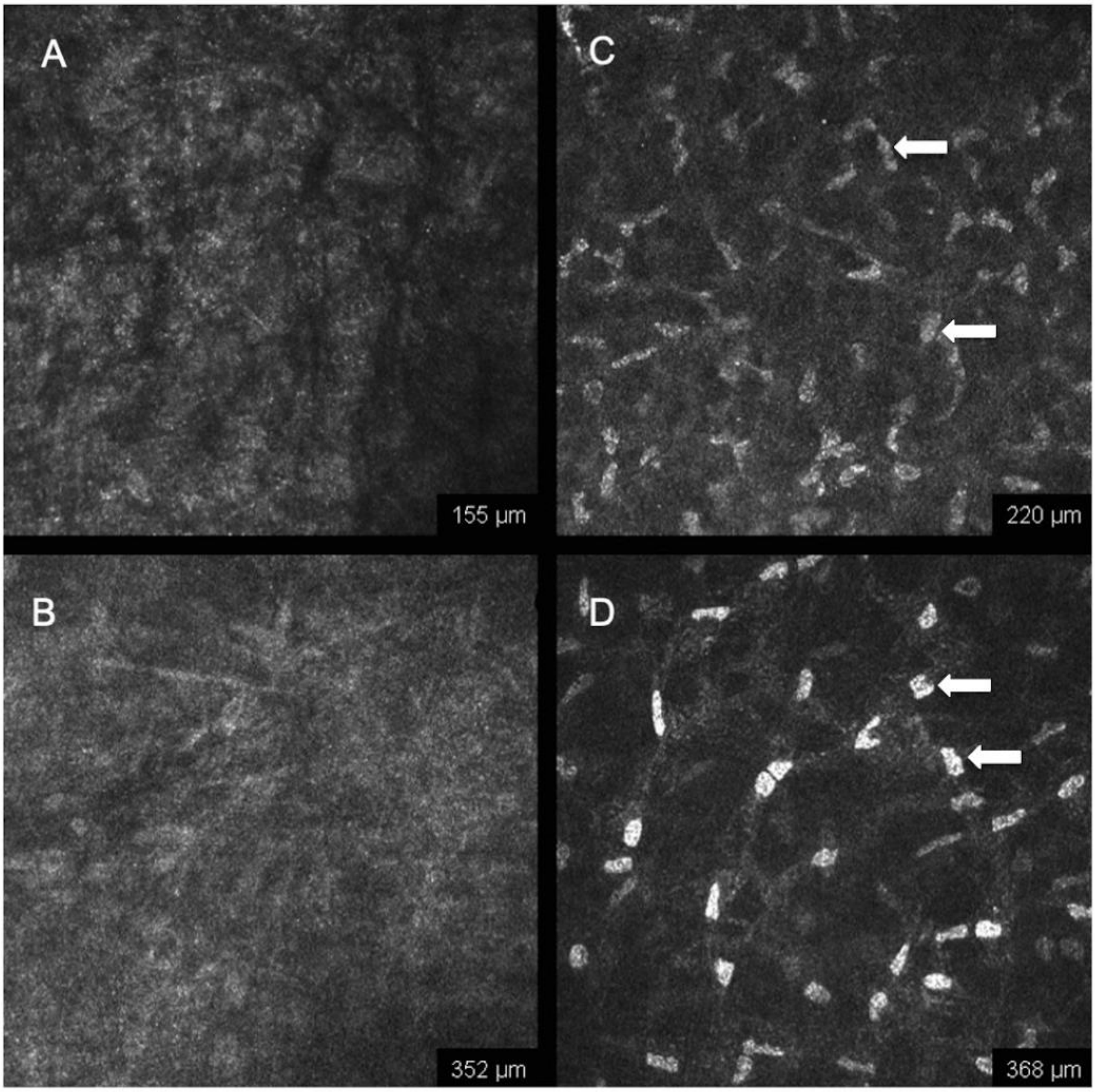

Supplementary Figure 1
Confocal microscopy image of the central corneal stroma of the left eye of patient III:1 (A, B) compared with scans of a normal eye (C, D). The images were captured in the superficial and mid-stroma. Images from the affected eye show extensive stromal remodelling with high interstitial matrix reflectivity, which obscures the keratocytes. In the scans from a normal cornea, the keratocytes can be visualised at both depths (arrows).

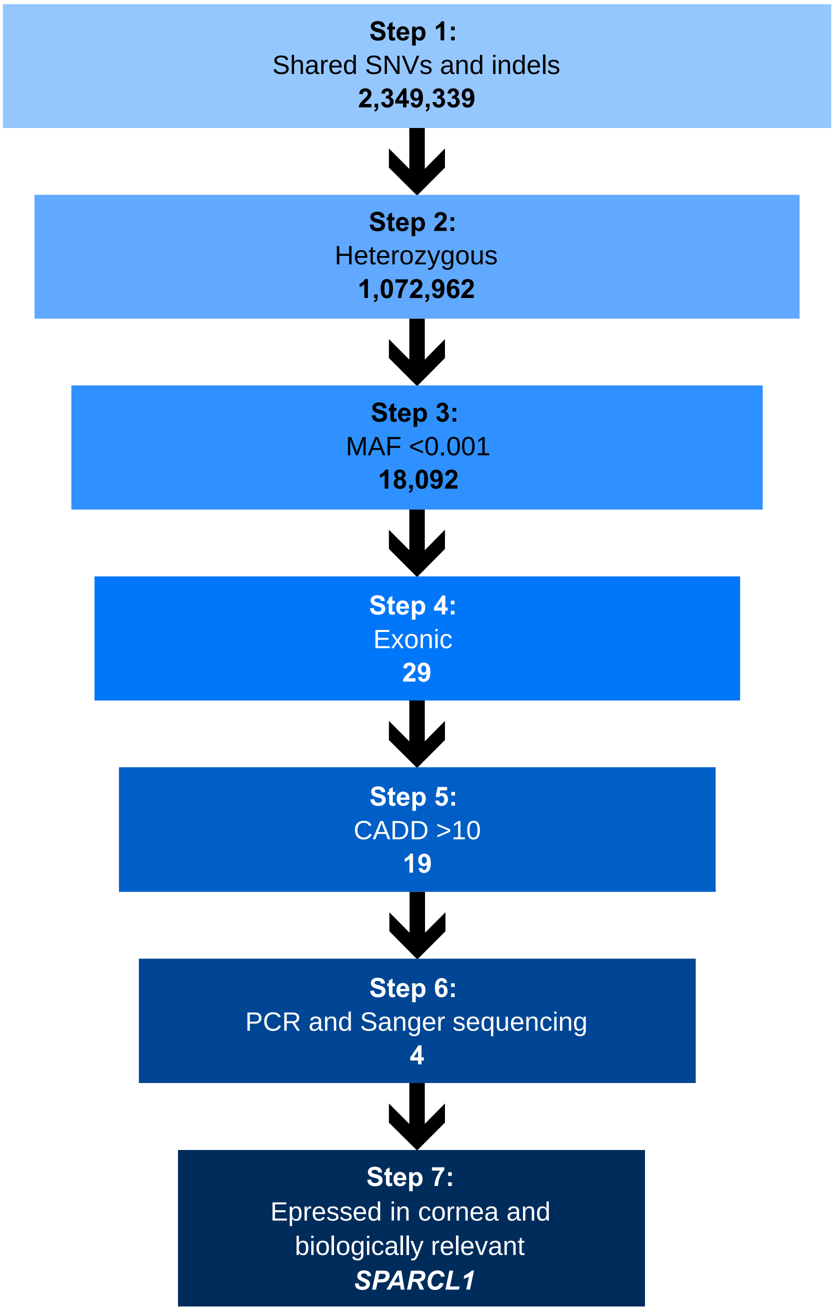

Supplementary Figure 2
The seven filtering steps applied the whole genome sequencing data of the four sequenced affected individuals (III:1, III:8, IV:1, IV:3). The list of SNVs and indels decreased from 2,349,339 variants to the 19 heterozygous, rare (MAF < 0.001), deleterious (CADD > 10) variants (Supplementary Table 3). Of these 19 variants, a variant in *SPARCL1* segregated with disease in the extended pedigree, is biologically relevant due to its reported regulation of decorin in the literature (1) and is expressed in the tissue of interest (Supplementary Fig. 3) (2,3).

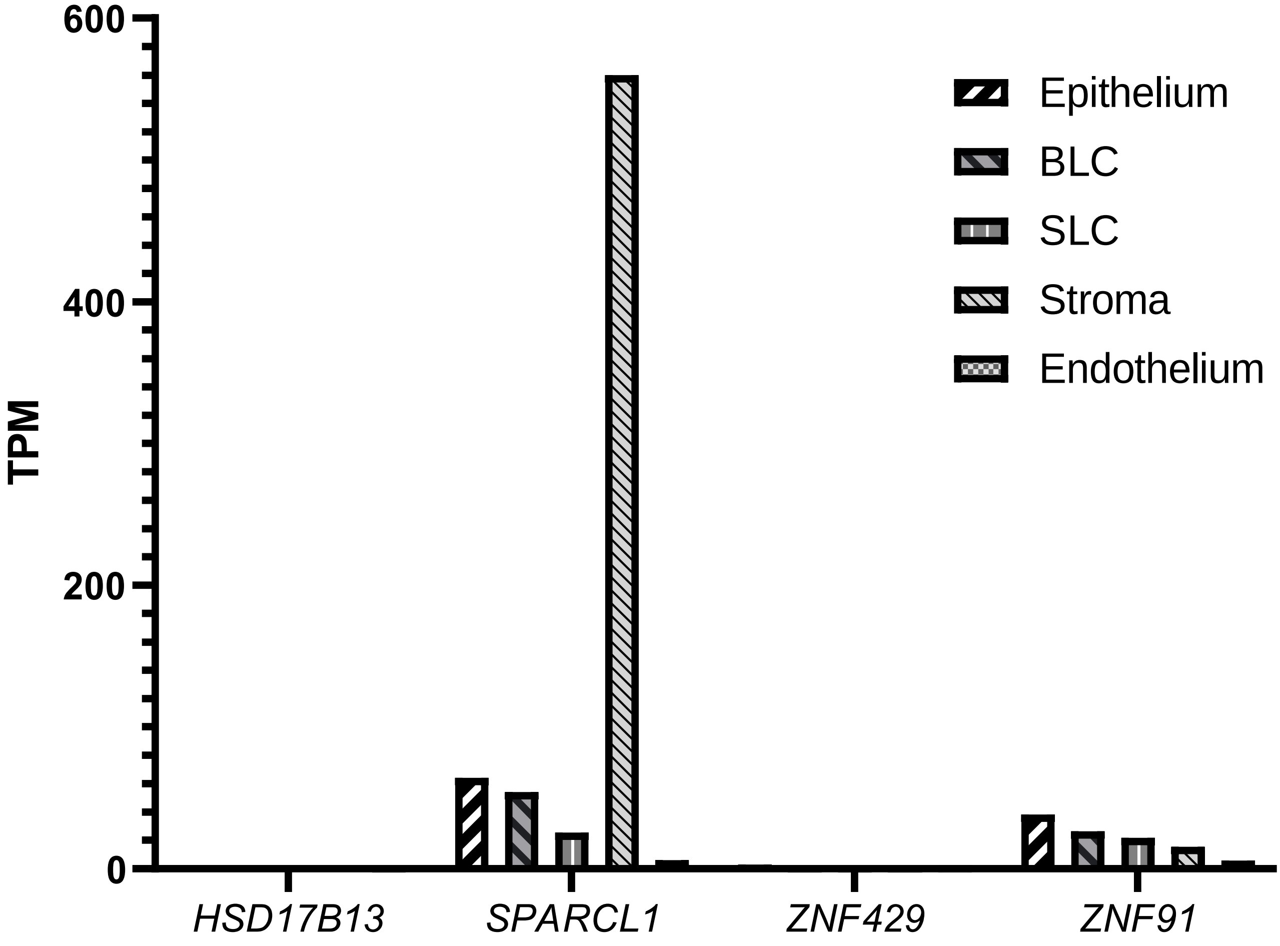

Supplementary Figure 3
RNA-seq transcript expression of the four candidate genes (*HSD17B13, SPARCL1, ZNF429, ZNF91)* in the different layers of the cornea. BLC basal limbal crypts; SLC superficial limbal crypts. Data curated from bulk RNA-Seq and presented as transcripts per million (TPM) (2,3).

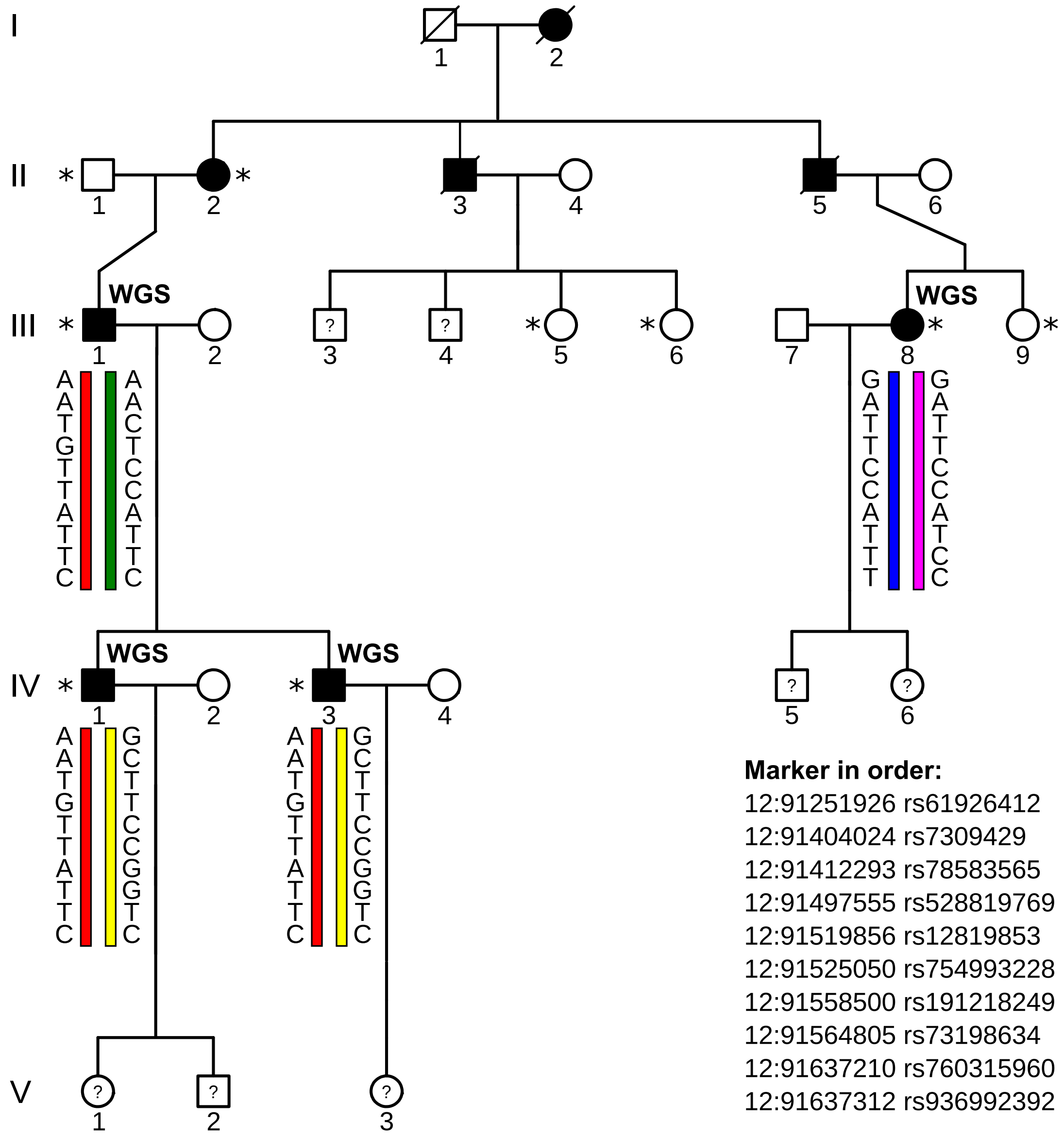

Supplementary Figure 4

Phased SNP/haplotype (chr12:91,251,926-91,637,312) encompassing *EPYC, KERA, LUM, DCN* using whole genome sequencing data of the four sequenced affected individuals (III:1, III:8, IV:1, IV:3) reveal no shared haplotype segregate with disease.

Gene	Mode of inheritance	Corneal Dystrophy
COL17A1	AD	Epithelial recurrent erosion dystrophy
KRT3, KRT12	AD	Meesmann corneal dystrophy
MCOLN1	AD	Lisch epithelial corneal dystrophy
TACSTD2	AR	Gelatinous drop-like corneal dystrophy
TGFBI	AD	Reis–Bücklers corneal dystrophy
TGFBI	AD	Thiel–Behnke corneal dystrophy
TGFBI	AD	Lattice corneal dystrophy, type 1 and its variants
TGFBI	AD	Granular corneal dystrophy, type 1
TGFBI	AD	Granular corneal dystrophy, type 2
CHST6	AR	Macular corneal dystrophy
UBIAD1	AD	Schnyder corneal dystrophy
DCN	AD	Congenital stromal corneal dystrophy
PIKFYVE	AD	Fleck corneal dystrophy
Deletion of *KERA*, *LUM*, *DCN*, and *EPYC*	AD	Posterior amorphous corneal dystrophy
PRDX3	AD	Punctiform and polychromatic pre-Descemet corneal dystrophy
COL8A2, TCF4, SLC4A11, ZEB1, AGBL1	AD	Fuchs endothelial corneal dystrophy
OVOL2, *ZEB1*, *GRHL2*	AD	Posterior polymorphous corneal dystrophy
SLC4A11	AR	Congenital hereditary endothelial dystrophy

Supplementary Table 1
This table summarises the genes known to be associated with various types of corneal dystrophies, their respective modes of inheritance and names of the corneal dystrophies they cause (4). AD autosomal dominant. AR autosomal recessive.

Gene	Forward primer (5’-3’)	Reverse primer (5’-3’)	Amplicon size (bp)	Annealing temperature (°C)
SPARCL1	CTTAGGCATAAATAT GGTTACATTATC	TTGGAAATATTTG GATCCTGC	511	60

Supplementary Table 2
This table presents the details of PCR primer pairs designed for amplifying specific regions within SPARCL1 exon 4 encompassing the variant of interest (c.334G>A).

Gene	Chr	Position	rsID	REF	ALT	Exonic Function	MAF
HSD17B13	4	88239500	rs540096704	ATCTCT	A	Frameshift deletion	0.00068
SPARCL1	4	88415618	.	C	T	Missense	0
ZNF429	19	21720222	.	A	G	Missense	0
ZNF91	19	23544979	.	A	G	Missense	0

Supplementary Table 3

This table lists genetic variants surviving Step 6 of the filtering strategy (Supplementary Fig. 2), including their associated gene names, chromosomal location (hg19), variant rsID (if available), reference (REF) and alternate (ALT) alleles, exonic functions, and MAF according to gnomAD v3.1.2. *HSD17B13*(NM_178135); *SPARCL1*(NM_001128310, NM_004684); *ZNF429*(NM_001001415, NM_001346912, NM_001346913, NM_001346914, NM_001346915, NM_001346916); *ZNF91*(NM_001300951, NM_003430).

References

1. Sullivan MM, Barker TH, Funk SE, Karchin A, Seo NS, Höök M, et al. Matricellular hevin regulates decorin production and collagen assembly. J Biol Chem. 2006 Sep 15; 281(37):27621–32.

2. Bath C, Muttuvelu D, Emmersen J, Vorum H, Hjortdal J, Zachar V. Transcriptional Dissection of Human Limbal Niche Compartments by Massive Parallel Sequencing. PLoS One. 2013 May 22;8(5):e64244.

3. Chen Y, Huang K, Nakatsu MN, Xue Z, Deng SX, Fan G. Identification of novel molecular markers through transcriptomic analysis in human fetal and adult corneal endothelial cells. Hum Mol Genet. 2013 Apr 4;22(7):1271.

4. Weiss JS, Rapuano CJ, Seitz B, Busin M, Kivelä TT, Bouheraoua N, et al. IC3D Classification of Corneal Dystrophies-Edition 3. Cornea. 2024 Apr 1;43(4):466–527.
